# Gene Inactivation in Transgenic Plants—A Unique Model for Studying Epigenetic Regulation of Gene Expression

**DOI:** 10.3390/plants15020247

**Published:** 2026-01-13

**Authors:** Tatyana V. Marenkova, Alla A. Zagorskaya, Igor V. Deyneko, Elena V. Deineko

**Affiliations:** 1 Laboratory of Plant Bioengineering Institute of Cytology and Genetics, Siberian Branch of Russian Academy of Sciences, 630090 Novosibirsk, Russia; zagorska@bionet.nsc.ru (A.A.Z.); deineko@bionet.nsc.ru (E.V.D.); 2 Department of Genetics and Breeding, Novosibirsk State Agrarian University, 630039 Novosibirsk, Russia; 3 Laboratory of Functional Genomics, K.A. Timityazev Institute of Plant Physiology of Russian Academy of Sciences, 127276 Moscow, Russia; igor.deyneko@inbox.ru

**Keywords:** transgene silencing, RNA-dependent DNA methylation, TGS, PTGS, epigenetic regulation, siRNA

## Abstract

The phenomenon of transgene silencing was first observed shortly after the generation of the initial transgenic plants. The vast body of experimental data accumulated since then constitutes an invaluable resource for dissecting the mechanisms of epigenetic gene regulation. Silencing operates at either the transcriptional (TGS) or post-transcriptional (PTGS) level and is predominantly mediated by small interfering RNAs (siRNAs). Although these two epigenetic pathways involve distinct sets of proteins and enzymes, they share fundamental mechanistic features: the generation of double-stranded RNA (dsRNA), its processing into siRNAs by DICER-LIKE (DCL) enzymes, and the assembly of an Argonaute-centered effector ribonucleoprotein complex (RISC). Guided by sequence-specific siRNAs, this complex identifies complementary target sequences with high precision. A comprehensive understanding of these regulatory pathways enables the targeted induction or suppression of specific plant genes. This review traces the history of experimental findings regarding the loss of recombinant gene activity in transformants and their progeny, which collectively established the foundation for elucidating the molecular mechanisms of transgene silencing.

## 1. Introduction

Genetically modified (GM) plants are indispensable tools for addressing a broad spectrum of fundamental and applied challenges, ranging from the elucidation of gene functions and the engineering of novel metabolic pathways to the development of resilient cultivars with enhanced nutritional profiles and resistance to biotic and abiotic stresses. Furthermore, GM platforms are increasingly utilized for the production of high-value recombinant proteins for therapeutic and industrial applications.

This progress has been underpinned by significant advancements in in vitro tissue culture, the refinement of transformation protocols, and the continuous innovation of genome engineering toolkits. Forty-two years after the generation of the first transgenic tobacco [1], plant transformation has become a routine procedure in molecular biology. However, achieving high-level transgene expression and ensuring its transgenerational stability remain persistent challenges. Notably, the seminal study by Barton et al. [1] already hinted at these complexities: the activity of the transferred nopaline synthase gene was observed in only 25% of self-pollinated progeny, significantly deviating from the expected 75% Mendelian ratio. The underlying causes of such discrepancies, obscure at the time, were only elucidated decades later through the discovery of epigenetic silencing mechanisms.

This review provides a comprehensive overview of the molecular pathways governing transgene silencing in plants. By synthesizing decades of experimental data and historical observations, we examine the fundamental mechanisms that were ultimately decoded at the molecular level, offering insights into modern strategies for stabilizing transgene expression.

## 2. Patterns of Transgene Inheritance and Expression Stability

**Figure 1:**
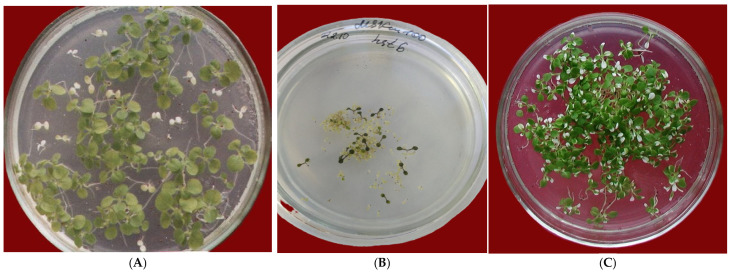
Patterns of stable and unstable *nptII* marker gene expression in transgenic plants. (**A**) Stable expression in tobacco. Self-pollination of a mono-insertional line yields a monohybrid segregation ratio (3 Km^+^:1 Km^−^) on selective medium. Green seedlings are kanamycin-resistant, while white seedlings are susceptible. (**B**,**C**) Transgene expression instability and non-Mendelian segregation. (**B**) Significant segregation distortion in T_1_ progeny of *Arabidopsis thaliana* L. (<1 Km^+^:1 Km^−^). An underrepresentation of resistant (green) progeny and an excess of susceptible (white) individuals are observed on selective medium. (**C**) Somatic mosaicism in transgenic tobacco line Nu21 [4]. Seedlings exhibit a variegated white-green phenotype under selection, indicating localized inactivation of the *nptII* gene in chlorotic leaf sectors. Images are provided by the authors.

The initial pioneering research on GM plant production established that introduced heterologous genes behave as dominant alleles, with their inheritance across generations conforming to classical Mendelian principles [1,2,3]. For example, self-pollination of a mono-insertional transgenic plant carrying the *nptII* marker gene (conferring resistance to kanamycin, Km^+^) yields progeny in the first generation (F_1_) that segregate in a monohybrid ratio of 3 Km^+^:1 Km^−^ (Figure 1A). In the case of two independent foreign DNA insertions, the segregation follows a dihybrid ratio of 15 Km^+^:1 Km^−^, and so forth. The Km^+^ progeny constitute the transgenic individuals, while the Km^−^ class consists of non-transgenic siblings. When crossing two independent transgenic plants, each possessing a single T-DNA insertion but carrying the same selective marker integrated into different genomic loci, the F_1_ generation will also segregate in a 3 Km^+^:1 Km^−^ ratio.

As previously noted, even pioneering studies on the stability and inheritance of heterologous genes in primary transgenic populations identified instances of non-Mendelian inheritance. As the number of characterized transgenic events increased, evidence of transgene expression instability continued to accumulate [5,6,7]. Numerous studies have reported that the segregation of progeny from self-pollinated, mono-insertional transformants on selective media often deviates from the expected 3:1 monohybrid ratio, yielding ratios such as 2:1, 1:1, or even <1:1 [8,9] (Figure 1B). In extreme cases, a complete loss of transgene expression has been observed [8,10]. In such instances, the proportion of resistant transgenic progeny decreases, while the sensitive phenotypic class expands to include both non-transgenic individuals and transgenic plants with silenced marker genes. Furthermore, expression instability can manifest as somatic mosaicism, characterized by alternating sectors of active and inactive transgene states within plant tissues [11]. This pattern of variable foreign gene expression has been documented across a wide range of marker and reporter genes, including *nptII* [4,12,13] (Figure 1C), *uidA* [14], chalcone synthase (*CHS*) [15], luciferase [16], and *gfp* [17].

Figure 1 presents original representative photographs from our laboratory’s long-term research on *Agrobacterium*-mediated plant transformation. In Figure 1A, seeds from self-pollinated T_0_ transgenic tobacco plants were germinated on a selective medium. These plants contain a stochastic integration of the *nptII* marker gene, driven by the CaMV 35S promoter. A classic monohybrid segregation pattern (3 Km^+^:1 Km^−^) is evident: resistant transgenic seedlings remain green, whereas susceptible non-transgenic seedlings exhibit a white, chlorotic phenotype. This ratio confirms the functional integration of a single T-DNA locus. In contrast, Figure 1B,C depict cases of transgene expression instability identified during primary screening. For instance, T_1_ progeny of *A. thaliana* L. transformants—carrying the *nptII* gene and the target gene homogentisate solanesyl diphosphate transferase—showed significant segregation distortion. An underrepresentation of kanamycin-resistant seedlings (<1 Km^+^:1 Km^−^) was observed (Figure 1B), precluding an accurate estimation of the transgene copy number. Figure 1C shows somatic mosaicism of the *nptII* marker gene in transgenic tobacco line Nu21. The chaotic alternation of green and white sectors across the leaf blade indicates localized inactivation of the marker gene during seedling development [4].

Loss of transgene expression in the T_1_ progeny of primary transformants is predominantly observed in lines harboring multiple T-DNA insertions integrated into one or more genomic loci [10,18,19]. While this inactivated state can persist through subsequent generations [4,7,14], rare instances of spontaneous expression restoration have also been reported [7,20]. Transgene silencing may also result from integration into hypermethylated genomic regions [21] or structural rearrangements within the genetic construct that compromise transgene integrity [8,22]. Additionally, expression loss may occur when integration occurs in proximity to endogenous genes involved in developmental regulation [23]. Finally, cases of transgene deletion in the progeny of primary transformants have been documented [8,22].

Observed deviations from Mendelian segregation in transgenic progeny may be confounded by genetic linkage. For instance, an analysis of GUS expression in F_2_ white clover hybrids revealed a shift from the expected 3 GUS^+^:1 GUS^−^ ratio to a 2 GUS^+^:1 GUS^−^ pattern. This deviation was attributed to an excess of heterozygotes rather than transgene inactivation. The authors proposed that the T-DNA integrated proximal to a recessive lethal allele; the occurrence of rare homozygous progeny was explained by meiotic recombination between the lethal locus and the T-DNA insertion [24]. Furthermore, transgene expression exhibits significant variability both among independent primary transformants and within their self-pollinated progeny. Notably, even when the *uidA* gene is targeted to the same chromosomal locus using the *Cre-loxP* site-specific recombination system, expression levels can vary drastically—ranging from stable high-level expression to mosaicism and complete silencing [14]. Environmental factors also modulate the stability of heterologous gene expression. Transitioning transformants and their progeny from controlled in vitro environments to field conditions can trigger substantial fluctuations in recombinant gene activity [25].

Thus, even during the early stages of data accumulation, it became evident that multiple factors contribute to the expression variability of recombinant genes and their non-Mendelian inheritance in transgenic plants. The majority of cases involving the loss of transgene expression in transformants and their progeny are not attributable to structural disruptions of the nucleotide sequence—such as deletions or mutations—but are instead driven by epigenetic modifications.

## 3. Homology-Dependent Gene Silencing: Conceptual Framework and Manifestations

The phenomenon of gene inactivation associated with multiple copies was termed homology-dependent gene silencing (HDGS). This mechanism refers to the silencing of genes (both transgenes and endogenous plant genes) triggered by the presence of several homologous sequences within the genome, which can share homology in the promoter region and/or the coding sequence. These multiple copies of exogenous DNA may reside in a single genetic locus or be dispersed across different genomic regions. HDGS operates at both the transcriptional level (transcriptional gene silencing, TGS) and the post-transcriptional level (post-transcriptional gene silencing, PTGS). Accordingly, TGS is characterized by the complete absence of mRNA transcripts. In contrast, PTGS involves the detection of mRNA in the nucleus, followed by its rapid degradation or translational arrest in the cytoplasm. Consequently, both mechanisms result in the failure of target protein accumulation.

### 3.1. Transcriptional Gene Silencing: Mechanistic Insights into Cis- and Trans-Inactivation

Transcriptional gene silencing can be further categorized into cis- and trans-inactivation (cis-TGS and trans-TGS), depending on the genomic localization of the transgenes. Cis-inactivation refers to the silencing of transgenes organized as clusters of multiple linked copies at a single locus [7,26]. In contrast, trans-inactivation involves the interaction between two unlinked transgenes integrated into different genomic loci. In this scenario, a transgene existing in an inactivated state induces the silencing of a previously active homologous transgene [27,28,29].

Cis-inactivation was initially documented in transgenic *A. thaliana* lines harboring multi-copy T-DNA insertions (5–10 copies) at a single locus, which correlated with the loss of *hpt* selectable marker expression. This silencing phenotype remained stable across multiple self-pollinated generations [7]. Similar silencing of antibiotic resistance was observed in *A. thaliana* lines containing *nptII* duplications within the T-DNA region during investigations into somatic and meiotic recombination frequencies [26,30]. In these studies, a construct featuring a direct *nptII* duplication flanking an *hpt* marker gene was utilized. The *nptII* copies contained non-functional 3′ and 5′ deletions, rendering the primary transformants resistant only to hygromycin. Among the self-pollinated T_3_ progeny, 22 kanamycin-resistant plants were identified, indicating the restoration of *nptII* integrity via homologous recombination. These recombinants represented an allelic series with varying *nptII* copy numbers at the same locus, ranging from a single intact copy to multiple duplications. Analysis of four subsequent generations demonstrated that stable expression was maintained only in single-copy lines, whereas tandem repeats led to the frequent segregation of antibiotic-sensitive progeny with varying frequency. A key advantage of this experimental model was the ability to assess the impact of transgene copy number on expression levels while controlling for position effects [26]. These findings led to the coining of the term repeat-induced gene silencing (RIGS) to describe inactivation triggered by tandem repeats within a construct. However, tandem repeats do not universally result in silencing; instances of stable expression in duplicated transgenes have been reported, sometimes leading to enhanced chimeric protein yields [31].

Trans-inactivation resulting from the interaction between multiple exogenous DNA copies was first identified in 1989 through double transformation experiments in tobacco [12]. The authors demonstrated that introducing the *hpt* marker gene (H construct) into the genome of a transgenic tobacco plant already carrying the *nptII* gene (K construct) resulted in the loss of the kanamycin-resistant phenotype in 15% of the double transformants. Expression of the *nptII* gene was restored only after the transgenes were segregated in the progeny of crosses with non-transgenic plants. Both constructs shared homologous regions, specifically two NOS promoters and the *nos* (nopaline synthase) gene sequence. A similar pattern of *nptII* inactivation in the presence of the *hpt* construct was observed when the two transgenes were combined through hybridization [32]. In these experiments, the *hpt* locus functioned as a silencer, while the *nptII* gene served as the target. Subsequent analysis of various H-locus alleles revealed that a complex, tandem T-DNA structure correlated with the ability to suppress genes driven by the NOS promoter. Notably, H-locus alleles that had undergone structural rearrangements, such as deletions within the *nos* gene region, lost their silencing capacity [33]. Furthermore, the efficiency of trans-inactivation was found to depend on the specific genomic localization of the T-DNA insertions. For example, re-transformation of four independent *hpt*-transgenic tobacco lines with an *nptII*-carrying T-DNA resulted in *hpt* silencing frequencies ranging from 1% to 10%, depending on the original transformant line [34].

Another compelling model of trans-inactivation is the tobacco transgenic locus 271, which harbors the *nptII* gene under the CaMV 19S promoter and the *RiN* gene (encoding an antisense RNA complementary to endogenous nitrate reductase *Nii* genes) driven by the CaMV 35S promoter [29]. In hybrids obtained from crosses with other transgenic lines, locus 271 behaved as a dominant silencer, inducing the inactivation of any transgene driven by either 19S or 35S promoters, irrespective of their genomic localization [29,35].

Trans-inactivation shares phenotypic similarities with paramutations—interallelic interactions where a paramutagenic allele induces directed, heritable, and reversible epigenetic changes in a homologous paramutable allele. Paramutagenic behavior was documented for the maize *A1* gene, which confers red flower coloration in transgenic petunia plants. Among homozygous transgenic individuals, lines with both white (17W) and red (17R) flowers were identified. In the F_1_ progeny of crosses between these lines, plants exhibited white or mosaic flower patterns, indicating that the 17W allele acted as a paramutagenic agent, leading to the partial or complete silencing of the 17R allele [28].

Collectively, these experimental findings demonstrate that the presence of homologous sequences within promoter regions is the primary determinant for triggering TGS mechanisms.

### 3.2. Post-Transcriptional Gene Silencing: Initiation, Systemic Propagation, and Epigenetic Resetting

The coordinated silencing of multiple transgene copies, or of transgenes and their homologous endogenous counterparts, driven by sequence homology within the coding region, is termed post-transcriptional gene silencing or co-suppression. This phenomenon was first characterized during efforts to intensify floral pigmentation in petunias by introducing additional copies of anthocyanin biosynthesis genes, specifically *CHS* and dihydroflavonol-4-reductase (*DFR*) [6,36]. Unexpectedly, approximately 25% of the resulting transformants exhibited white or variegated flowers, signaling the concurrent silencing of both the transgenes and the native host genes. Subsequent investigations established that this suppression results from targeted mRNA degradation within the cytoplasm [37]. Notably, locus 271, previously described as a mediator of transcriptional silencing, also triggers PTGS of endogenous *Nii* genes [29]. Similar co-suppression mechanisms have been documented across various species, including tobacco, petunia, and *A. thaliana*, affecting diverse gene families such as β-1,3-glucanase [38], β-glucuronidase [39], chitinase [40], S-adenosyl-L-methionine synthetase [41], nitrate reductase [42], and others.

The co-suppression mechanism is categorized into three distinct stages: initiation, propagation, and maintenance of the silenced state. The probability of PTGS in transgenic plants is multifactorial, depending on the architecture and copy number of foreign insertions, the magnitude of gene expression, the developmental stage of the plant, and environmental variables. Silencing occurs more frequently in transformants that are homozygous for the transgene locus, harbor multi-copy insertions, or utilize strong promoters that drive high transcript levels. Conversely, the formation of truncated small transcripts or the complete inhibition of transcription prevents the onset of PTGS. Efficient triggering of PTGS is also strongly associated with the presence of inverted or direct tandem repeats within the insertion. For example, two direct duplications fused into a single cistron resulted in silencing frequencies of 80–100%, while increasing the repeat number to three or four led to a complete loss of transgene expression. This dosage-dependent effect of copy number at a single locus has been demonstrated across diverse markers, including *uidA*, *GFP*, *CAT* (chloramphenicol acetyltransferase), and *CAB1* (chlorophyll a/b-binding protein) [43]. Furthermore, the timing and frequency of co-suppression initiation vary significantly with light intensity and growth conditions, ranging from in vitro cultures to greenhouse and field environments [36,44].

The subsequent stage—the propagation of coordinated silencing across the whole plant—is characterized by the progressive manifestation of altered phenotypic traits. Notable examples include leaf chlorosis in tobacco following the silencing of nitrate reductase genes [44] and the varying degrees of white or variegated floral pigmentation in petunia resulting from *CHS* and *DFR* suppression [6,36]. The systemic spread of PTGS is facilitated not only by the mitotic transmission of the silenced state to daughter cells but also by long-distance intercellular communication. Grafting experiments have demonstrated the transmission of PTGS induction signals between tissues with different heterologous gene expression levels. For instance, grafting scions with silenced *Nia*, *Nii* [42], or *gfp* [45] genes onto rootstocks exhibiting high transgene activity triggered systemic silencing throughout the entire plant. These experiments also confirmed that PTGS is stably maintained in transformants harboring a transgene capable of both initiating and propagating the silencing signal. In contrast to transcriptional cis- and trans-inactivation, co-suppression is generally not inherited through meiosis. Epigenetic resetting occurs during early embryogenesis, leading to the restoration of gene expression. For example, while β-1,3-glucanase silencing in transgenic tobacco remained stable during vegetative growth, flowering, and in vitro shoot regeneration, in situ hybridization revealed that expression was restored in embryonic tissues post-fertilization. Consequently, the silencing process must be re-initiated in the progeny, typically manifesting 3–4 weeks after germination [46].

In summary, co-suppression represents a highly coordinated and dynamic regulatory mechanism triggered by coding sequence homology, governing transgene and endogenous gene expression through a sophisticated interplay of initiation, systemic signaling, and developmental resetting.

## 4. Molecular Genetic Mechanisms of Exogenous DNA Inactivation in the Genome of Transgenic Plants

The silencing of heterologous gene expression can occur both within tightly linked tandem copies at a single genetic locus and among copies dispersed across different chromosomal regions. This observation raises a fundamental question: Are there structural or positional differences between transgene loci that initiate silencing (silencers) and those that are susceptible to it (targets)? Table 1 summarizes research data on five characterized silencer loci (H59, H9np, H11, H2, and 271), one silencing-susceptible locus (K81), and several relatively silencing-resistant counterparts (H9, Kα and H83) in model transgenic tobacco plants.

The integration of one or two intact T-DNA copies into telomeric, transcriptionally active euchromatic regions—combined with the absence of vector backbone sequences—correlates with stable transgene expression and diminished sensitivity to silencing mechanisms in tobacco lines Kα, H9, and H83. In contrast, the presence of multi-copy, rearranged insertions, inverted duplications, and vector-derived sequences, particularly when integrated into intercalary heterochromatin or pericentromeric regions, triggers de novo DNA methylation and expression instability in lines H11, H59, H2, H9np, and 271. These lines exhibit “silencer” properties, enabling them to suppress the expression of other homologous transgenes in *trans*. Notably, target genes vary in their epigenetic susceptibility: lines Kα, H9, and H83 remain resistant, whereas line K81 is highly sensitive to trans-inactivation. Consequently, the structural architecture and copy number of the T-DNA insertion, the co-integration of vector backbone sequences, and the specific chromosomal environment collectively determine transgene expression levels and their vulnerability to homology-dependent silencing.

It is hypothesized that integration into gene-rich subtelomeric regions promotes stable transgene expression, whereas proximity to pericentromeric or subtelomeric repeats and intercalary heterochromatin triggers expression instability. An analysis of 973 independent *A. thaliana* transformants revealed that T-DNA integration occurred within centromeric, telomeric, or ribosomal repeats in only 4.7% of cases, while 35.4% of insertions targeted coding sequences, and the remainder localized to intergenic regions [50]. Furthermore, a large-scale mapping of 88,122 independent T-DNA integration sites across the five *A. thaliana* chromosomes identified “hotspots” with a distinct preference for foreign DNA integration. The authors established a significant integration bias, with the lowest insertion frequency observed in pericentromeric regions and the highest in gene-dense chromosomal areas [51]. Functional analysis of a reporter gene integrated into various chromosomal loci, conducted in a TGS-deficient mutant background, confirmed the existence of a global epigenetic network. This network coordinately regulates gene expression based on both the chromosomal environment and the specific nucleotide sequence of the target gene [52].

### 4.1. Molecular Pathways of Transcriptional Gene Silencing: The RdDM Framework

For nearly two decades, the scientific community focused on accumulating, describing, and systematizing experimental data regarding the loss of recombinant gene activity across various plant species. This foundational work set the stage for the next phase of research: the molecular elucidation of transgene silencing. Transgenic plants with inactivated exogenous DNA proved to be unique and powerful models for investigating the molecular pathways of gene silencing. Within these undesirable events of expression loss lie grains of gold—the discovery of previously unknown mechanisms of gene inactivation in the plant genome, along with the identification of novel proteins and enzymes, some of which are unique to the plant kingdom. Subsequent research has predominantly utilized *A. thaliana* as a model organism, owing to its rapid life cycle, compact size, and, most crucially, its fully sequenced and annotated genome and extensive mutant collections [53]. Through induced mutagenesis and the screening for monogenic mutations that restored the expression of previously silenced transgenes, dozens of proteins involved in silencing mechanisms were identified and mapped. Conversely, mutations that promoted the silencing of heterologous genes were also characterized; these findings suggest that the function of the corresponding wild-type alleles is to maintain an active transcriptional state even in the presence of repressive epigenetic marks.

Transcriptional gene silencing is mediated by epigenetic modifications, including cytosine methylation within promoter regions and specific histone modifications. The most extensively characterized mechanism is RNA-directed DNA methylation (RdDM), which was discovered during studies on *Potato spindle tuber viroid* (PSTVd) replication in transgenic tobacco plants harboring viroid cDNA in their nuclear genome [54]. Autonomous RNA replication cycles were shown to trigger site-specific cDNA methylation, a process linked to the formation of double-stranded RNA (dsRNA) subsequently processed into small RNAs [55]. Experiments using transgenic tobacco lines carrying various PSTVd cDNA fragments established that the minimum target sequence for RdDM is 30 bp [56]. RdDM is a sophisticated regulatory system involving not only the canonical RNA polymerase II but also two plant-specific polymerases: Pol IV and Pol V. The primary requirement for generating small interfering RNAs (siRNAs) that direct these epigenetic modifications is the presence of dsRNA. RNA Pol IV transcribes non-coding RNA from the target DNA, which is converted into dsRNA by RNA-dependent RNA polymerase 2/6 (RDR2/6). This dsRNA is then cleaved by Dicer-like 3 (DCL3) into 24 bp siRNAs, which are methylated at their 3′ ends by the methyltransferase HUA ENHANCER 1 (HEN1) and loaded into specialized ribonucleoprotein complexes containing Argonaute proteins, primarily AGO4. Within these complexes, the single-stranded guide siRNA identifies complementary sequences, subsequently recruiting the enzymes responsible for DNA methylation and repressive histone modifications. These coordinated processes occur within the cell nucleus in specialized subnuclear compartments.

**Figure 2:**
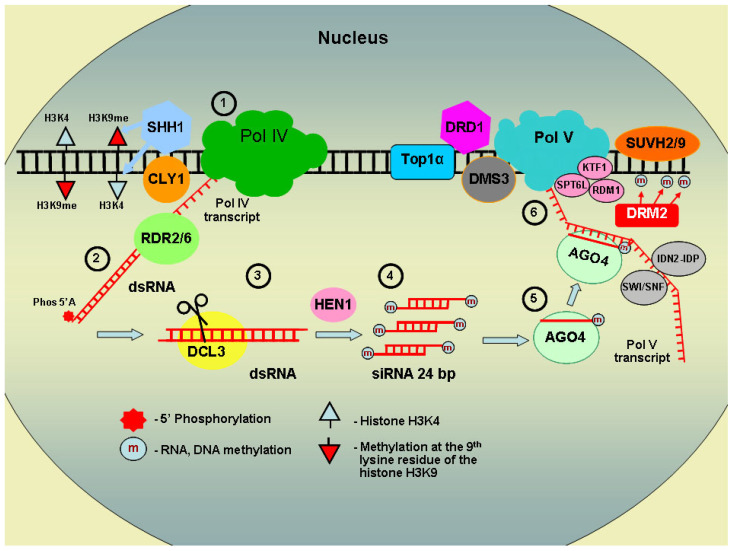
The canonical RdDM pathway. *Initiation and dsRNA Synthesis*: (1) Pol IV transcribes non-coding RNAs from target loci; its recruitment is mediated by SHH1 and the SWI2/SNF2-like factor CLSY1. SHH1 facilitates this by recognizing H3K9me and unmethylated H3K4 histone marks. (2) Pol IV transcripts are converted into dsRNA by RDR2/6. *Primary siRNA Biogenesis*: (3) dsRNA is processed by DCL3 into 24 bp siRNAs, (4) which are subsequently methylated at their 3′ ends by HEN1. *Effector Phase*: (5) These siRNAs are loaded into AGO4-containing complexes, retaining a single-stranded guide RNA. (6) Pol V, recruited by SUVH2 and SUVH9, generates scaffold transcripts that act as a docking platform for siRNA-AGO4 complexes. This assembly recruits DRM2 to catalyze DNA methylation. Access to the DNA template is ensured by TOP1α and the DDR complex (DRD1, DMS3, RDM1), while KTF1 acts as an elongation factor. The IDN2–IDP complex stabilizes the siRNA–scaffold duplex and, together with SPT6L and the SWI/SNF complex, modulates nucleosome positioning and facilitates DRM2 activity.

Let us examine this gene silencing mechanism at the transcriptional level in more depth (Figure 2). In plants, the canonical RNA polymerases (Pol I, Pol II, and Pol III) are supplemented by two specialized isoforms, Pol IV and Pol V, which evolved from Pol II and are essential for RdDM [57]. Pol IV transcribes relatively short non-coding RNAs (26–45 nt) from target DNA sequences, which are subsequently converted into dsRNA by RDR2 or RDR6. Unlike Pol II products, Pol IV transcripts lack a 5′ cap, a 3′ poly(A) tail, and introns. Instead, they possess 3′ overhangs and a 5′ monophosphate, suggesting the involvement of an unidentified phosphatase or inherent phosphatase activity within the Pol IV complex [58]. Pol IV predominantly targets intergenic regions, as well as euchromatic genes associated with repeats and transposons, accounting for approximately 90% of all 24-nt siRNA-producing loci [59]. The recruitment of Pol IV to target sites is mediated by SAWADEE HOMEODOMAIN HOMOLOG 1 (SHH1) and the SWI2/SNF2-like factor CLASSY 1 (CLSY1). SHH1 interacts with the polymerase and simultaneously recognizes H3K9me and unmethylated H3K4 histone marks via its unique tandem Tudor-like domain (with two ‘pockets’) [60]. Interestingly, SHH1, in association with METHYL-CpG-BINDING DOMAIN 7 (MBD7) and ALPHA CRYSTALLIN DOMAIN (ACD) proteins, may also function to enhance the expression of certain methylated genes [61]. CLSY1 associates with Pol IV through a conserved CYC-YPMF motif on the complex exterior, facilitating chromatin remodeling and the maintenance of tissue-specific methylation patterns [62,63,64]. Furthermore, RdDM at low-copy loci may require Pol II-derived non-coding transcripts to initiate the amplification of Pol IV-dependent siRNAs, thereby facilitating the trans-silencing of unlinked homologous genomic sequences [65].

During the subsequent stage, dsRNA is cleaved by the ribonuclease DCL3 into 24 bp siRNAs. These duplexes undergo 3′ end methylation by the HEN1, a modification that likely protects them from degradation or further enzymatic processing [66]. The resulting siRNAs are then loaded into specialized ribonucleoprotein complexes containing Argonaute proteins, primarily AGO4 or AGO6. Upon loading, the passenger strand is discarded, while the antisense guide strand directs the effector complex to the target gene via sequence complementarity [67]. At this junction, Pol V performs a critical function by generating non-coding scaffold transcripts, approximately 200 nt in length. These transcripts are proposed to be retained on chromatin by the RRP6-like protein RRP6L1 [68], serving as a recruitment platform for siRNA-AGO complexes and facilitating the assembly of enzymes responsible for DNA and histone methylation. Effective Pol V transcription requires Topoisomerase 1α (TOP1α) [69] and the DDR complex, which comprises DEFECTIVE IN RNA-DIRECTED DNA METHYLATION 1 (DRD1), involved in chromatin remodeling; DEFECTIVE IN MERISTEM SILENCING 3 (DMS3), a protein homologous to conserved SMC proteins; and RNA-DIRECTED DNA METHYLATION 1 (RDM1), a small, plant-specific protein [70,71]. These regulatory factors ensure Pol V access to single-stranded DNA. Furthermore, the conserved Pol II elongation factor SPT6-like (SPT6L) has been identified as an essential component for maintaining the abundance and integrity of Pol V scaffold transcripts [72,73].

Pol V interacts via the carboxyl-terminal domain (CTD) of its largest subunit, NRPE1, with both AGO4 and the elongation factor KTF1 (KOW domain-containing transcription factor 1), which similarly possesses an AGO4-binding motif [74]. Current models propose that siRNAs within the AGO4 effector complex bind complementarily to Pol V-generated scaffold transcripts, thereby recruiting the de novo DNA methyltransferase DRM2 to the target locus. The RDM1 protein, a component of the DDR complex, may further facilitate DRM2 recruitment through its dual interaction with AGO4 and DRM2 and its ability to bind single-stranded methylated DNA [70]. The IDN2–IDP complex (involved in De Novo 2 and its paralogs IDP1/IDP2) stabilizes the siRNA–scaffold RNA duplex and modulates nucleosome positioning through interactions with the SWI/SNF chromatin-remodeling complex, while also influencing DRM2 binding to the Pol V transcript. Notably, IDN2 association with these non-coding transcripts has been shown to be AGO4-dependent [74]. Although the precise mechanisms by which Pol V identifies its target sequences remain an area of active research, it is established that Pol V predominantly localizes to transposons and repetitive elements within introns, promoters, and coding regions [75]. Pol V recruitment to these methylated sites is mediated by the SUVH2 and SUVH9 histone methyltransferase homologs. Despite lacking catalytic activity, these proteins utilize their SRA (SET- and RING-associated) domains to bind methylated DNA and interact with the DDR and MORC (Microrchidia-type ATPase) complexes, the latter of which regulates higher-order chromatin architecture [76,77].

RNA splicing factors have emerged as integral components of the RdDM pathway in *A. thaliana*. Specifically, the arginine/serine-rich protein SR45 is active during the early stages of siRNA biogenesis, while ZOP1 (zinc-finger and OCRE domain-containing protein) is thought to modulate either the AGO4 effector complex or DRM2 activity. The PRP6-like splicing factor STA1 affects both siRNA production and the generation of Pol V scaffold transcripts, and the U4/U6 snRNP-associated protein RDM16 has been identified as a regulator of Pol V transcript abundance [78]. Additionally, the SNF2-ring-helicase-like proteins FRG1 and FRG2, which physically interact with SUVR2, are essential for efficient RNA-directed DNA methylation [79]. For a long time after its discovery, the function of the MOM1 (Morpheus’ molecule 1) protein was unknown. The *mom1* mutation was originally identified by its capacity to restore the expression of silenced multi-copy transgenes and endogenous repetitive elements. Crucially, this transcriptional reactivation occurs without altering canonical heterochromatic hallmarks, such as DNA hypermethylation or repressive histone modifications (e.g., H3K9 methylation and depleted H3K4 methylation) [80]. Recent evidence indicates that MOM1 participates in RdDM-mediated silencing for a subset of transgenic and endogenous targets. By binding to these loci, MOM1 facilitates interactions with the MORC6 protein [81], which in turn promotes Pol V function and the subsequent initiation of target de novo DNA methylation [82].

### 4.2. Non-Canonical RdDM Pathways

In addition to the canonical RdDM mechanism involving RNA polymerase IV, RDR2, DCL3, RNA polymerase V, AGO4, and 24-nt siRNAs, other cases of gene repression are known. These involve components of post-transcriptional gene silencing, specific proteins, and other types of small RNAs. For instance, in rice (*Oryza sativa*), it has been shown that RNA in the form of a hairpin can be cut by the enzyme DCL3, forming a long microRNA of 24-nt (lmiRNA). This microRNA is loaded into AGO4 and can direct methylation of the target nucleotide sequence [83]. Non-canonical RdDM primarily targets the repression of transposable elements (TEs) in the plant genome and is associated with Pol II transcription and RDR6 to generate 21–22-nt siRNAs, which, in complex with AGO6, direct TEs methylation [84].

### 4.3. The Epigenetic Landscape of TGS: DNA Methylation and Histone Crosstalk

Cytosine methylation is a conserved and universal mechanism for regulating gene expression in both mammals and plants. However, unlike in mammals, DNA methylation in plants occurs across three distinct sequence contexts: symmetric (CG and CHG) and asymmetric (CHH, where H = A, C, or T). These modifications are established and maintained by three classes of cytosine methyltransferases. The MET1 (METHYLTRANSFERASE 1) family is responsible for the maintenance of CG methylation. Plant-specific chromomethylases, CMT2 and CMT3, mediate the maintenance of CHH/CHG and CHG methylation, respectively, while the DRM (DOMAINS REARRANGED METHYLTRANSFERASE) family facilitates de novo methylation. Methylation in the CHG context is mechanistically coupled with the dimethylation of histone H3 lysine 9 (H3K9me2), catalyzed by the histone methyltransferases KYP/SUVH4, SUVH5, and SUVH6. These enzymes bind to methylated CHG sites to modify histones; conversely, CMT3 recognizes H3K9me2 marks to catalyze subsequent CHG methylation. This interaction establishes a self-reinforcing epigenetic loop that maintains stable gene repression [85]. Unlike symmetric contexts, the maintenance of asymmetric CHH methylation following DNA replication requires a persistent trigger signal, intrinsically linking it to the RdDM pathway. In specific regions of the *A. thaliana* genome, CHH methylation can be maintained through two independent pathways: either via the canonical RdDM mechanism or through the activity of CMT2 [86].

Transcriptional silencing often initiates with the loss of tissue-specific transgene expression patterns. For example, in transgenic rice harboring *uidA* insertions, certain homozygous T_1_ individuals exhibited a loss of β-glucuronidase activity specifically in phloem tissues, which correlated with enhancer methylation within the promoter. By the T_2_ generation, complete transgene silencing across all tissues was observed, accompanied by the spreading of cytosine methylation from the enhancer to both the promoter and the transcribed regions [87]. To identify the primary trigger of this process, a methodology utilizing the *RUBY* reporter for real-time visual assessment was developed in *A. thaliana* and lettuce (*Lactuca sativa*). This approach, combined with comprehensive transcript profiling, demonstrated that the production of aberrant RNAs—resulting from mRNA cleavage during ribosomal translation—precedes the formal initiation of RNA interference [88].

A fundamental unresolved question remains whether DNA methylation is the cause or a secondary consequence of gene inactivation. Notably, instances of methylated yet transcriptionally active genes have been documented [89,90]. Furthermore, the use of mutant 35S promoter variants lacking symmetric cytosine methylation sites did not preclude silencing when combined with the silencer locus 271 [91]. Additionally, several mutations have been identified that reactivate silenced transgenes without altering their methylation status [92,93]. Collectively, these experimental data suggest that while methylation may not be a strictly necessary condition for the initiation of silencing, it is essential for the stable maintenance of the repressive epigenetic state.

The methylated state of cytosine, as a stable DNA modification, can be inherited across subsequent generations [5,32,89]. Such transgene epimutations may persist through multiple generational cycles [7,32,94,95] or undergo reversion during plant development or upon backcrossing with wild-type individuals [5,7,94]. This instability can result in mosaicism, where a single plant harbors cell lineages with divergent epigenetic states—active and inactive [4,12,87,94]. In trans-inactivation experiments, mosaicism was observed following the segregation of K- and H-transgenes in the F_1_ generation (K/H x w.t.). Progeny with the K/− genotype exhibited variegated white-green leaf patterns on kanamycin-selective media. The white sectors were associated with the maintenance of a silenced *nptII* state, sustained by promoter hypermethylation, whereas expression was restored in green sectors. Following one or two additional generations, the hypomethylated state of the marker gene was typically fully restored, leading to uniform green coloration [96].

In plants, DNA demethylation occurs not only through passive dilution but also via active, replication-independent pathways mediated by the DEMETER (DME) family of DNA glycosylases, which includes DME, REPRESSOR OF SILENCING 1 (ROS1), DML2, and DML3 [97]. The antagonistic balance between siRNA-directed DNA methylation and active demethylation allows plants to dynamically modulate their genomic modification landscape in response to internal developmental cues and external environmental signals [98]. This reciprocal relationship is further supported by evidence that many core RdDM components were originally identified as suppressors of the *ros1* mutation (e.g., *rpa2*, *tsl1*, *rdm2*, *ago6*, *sup32*, *ctf1*). Conversely, mutations in key silencing genes—including *ago6*, *ago4*, *drm2*, *rdr2*, *drd1*, *met1*, and those encoding subunits of Pol IV and Pol V—result in the significant downregulation of *ROS1* expression [99].

The foundational model of gene silencing has been primarily established using *A. thaliana*, tobacco, and petunia. In *A. thaliana*, RdDM-deficient mutants remain viable and fertile, facilitating extensive genetic dissection of these pathways. However, in other plant species, mutations in the RdDM machinery often result in severe pleiotropic effects, including lethality, compromised fertility, genomic instability, and heightened pathogen susceptibility. Significant evolutionary divergence in these mechanisms has been observed. For instance, in conifers such as *Pinus contorta*, the 24 nt siRNA class and *DCL3* homologs are absent; instead, silencing is mediated by a diverse population of 21 nt small RNAs and a novel *DCL* family [100]. Conversely, in Chinese fir (*Cunninghamia lanceolata*), 24 nt siRNA-dependent RdDM and *DCL3* activity have been confirmed [101]. Furthermore, an analysis of *Brassica rapa* mutants deficient in the largest subunit of Pol IV revealed a substantially lower proportion of Pol IV-dependent siRNAs compared to *A. thaliana* [102]. In the Rosaceae family, most methylation maintenance and demethylation enzymes have been identified, along with core RdDM components including Pol IV (*NRPD1*), Pol V subunits (*NRPE1*), *RDR2*, *RDR6*, *AGO4*, *AGO6*, *DCL3*, and *DRM2* [103].

### 4.4. Post-Transcriptional Gene Silencing: Molecular Drivers

**Figure 3:**
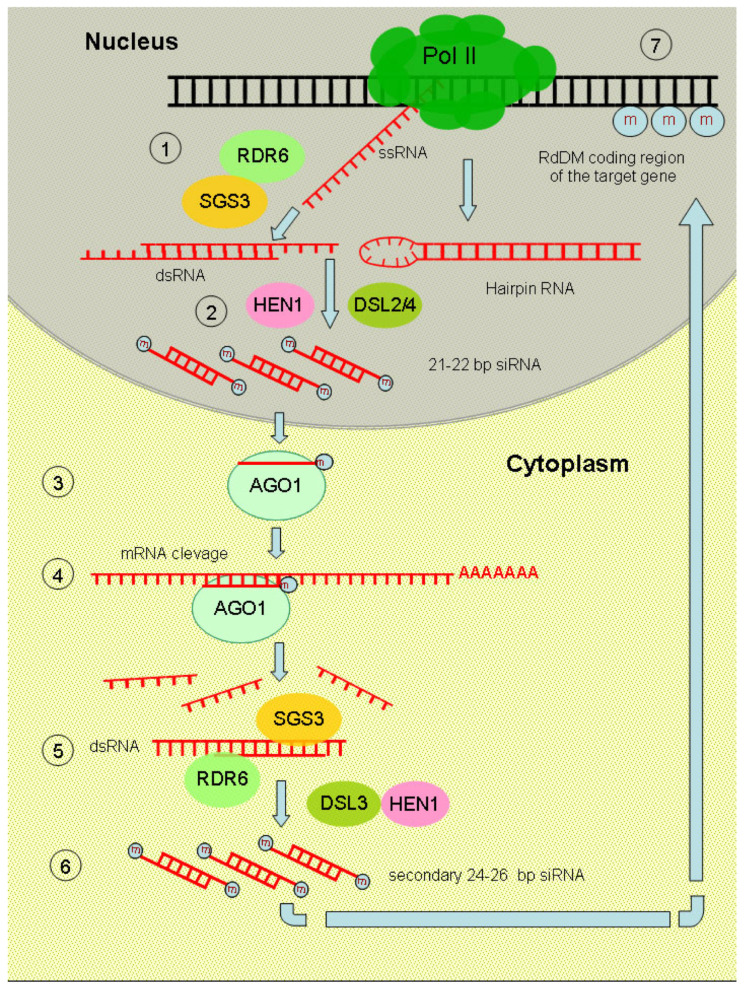
PTGS pathway. *Initiation and dsRNA Synthesis*: (1) Pol II transcribes single-stranded RNA (ssRNA). This RNA is converted into dsRNA by RDR6 in coordination with the coiled-coil protein SGS3, which stabilizes the RNA template. Alternatively, inverted repeats can be transcribed to form self-complementary hairpin structures. *Primary siRNA Biogenesis*: (2): The dsRNA is cleaved by DCL2/4 into 21–22 bp siRNAs. These siRNAs are subsequently stabilized by 3′-end methylation catalyzed by the methyltransferase HEN1. *Effector Phase*: (3–4) The siRNAs are loaded into an AGO1-containing RISC (RNA-Induced Silencing Complex) in the cytoplasm. The single-stranded guide siRNA directs the AGO1 complex to recognize and enzymatically cleave (slicing) complementary target mRNAs. *Secondary siRNA Biogenesis (Amplification)*: (5–6) The resulting mRNA cleavage products (aberrant RNAs) are recognized as substrates for secondary siRNA production. They are converted back into dsRNA by the RDR6–SGS3 complex. DsRNA is processed by DCL3 into 24–26 bp secondary siRNAs, which are subsequently methylated at their 3′ ends by HEN1. *Systemic Silencing and Chromatin Feedback*: (7) These secondary siRNAs can trigger the RdDM pathway, leading to de novo DNA methylation of the coding regions of target genes, thereby bridging post-transcriptional silencing with transcriptional repression.

PTGS was initially identified in transgenic petunia during efforts to modify floral pigmentation by overexpressing the anthocyanin biosynthesis gene *CHS*. Unexpectedly, this resulted in white or variegated purple-white flowers, signaling the concurrent silencing of both transgenes and their endogenous counterparts [6]. PTGS occurs primarily in the cytoplasm and involves the targeted degradation or translational repression of gene-derived mRNA (Figure 3). This sequence-specific degradation is directed by 21–22 nt siRNAs derived from double-stranded RNA precursors. These dsRNAs originate either from RNA polymerase II-driven transcripts of inverted repeats that form stable hairpin structures or from single-stranded mRNAs converted into dsRNA by RDR6 in coordination with the coiled-coil protein SGS3 (SUPPRESSOR OF GENE SILENCING 3).

The ribonuclease DCL4 cleaves these dsRNAs into 21–22 bp siRNAs, which undergo 3′ end methylation by the methyltransferase HEN1. The core PTGS effector complex, localized in the cytoplasm, utilizes the AGO1 protein as its catalytic component. During siRNA loading, the passenger strand is discarded, and the antisense guide strand directs the AGO1 complex to enzymatically hydrolyze complementary mRNAs [104]. Concurrent with mRNA degradation, de novo DNA methylation often occurs within the coding regions of target genes; although this typically does not block transcription, it may serve to stabilize the silenced state. These epigenetic modifications are directed by secondary 24–26 nt siRNAs produced via DCL3 activity. DNA methylation in the coding region is proposed to regulate post-transcriptional expression by disrupting RNA synthesis, specifically by inhibiting transcript elongation and promoting the formation of aberrant RNA species that act as substrates for silencing signal amplification. Studies utilizing recombinant Potato Virus X (PVX) as a vector have revealed a direct correlation between RNA sequence length and its capacity to trigger PTGS and methylation of homologous transgenes. It was established that the minimum threshold for perfect sequence identity is 23 nt, which corresponds to the typical size of small RNAs associated with PTGS in plants [105]. However, stable induction of PTGS generally requires more extensive homology, typically ranging from 300 to 600 nt. While experiments in RNAi vector design have shown that sequences as short as 50 nt can initiate silencing, their efficiency is significantly lower compared to longer fragments [106].

A separately identified mechanism is virus-induced gene silencing (VIGS), and viral suppressor proteins that block different stages of silencing are known [107].

Table 2 summarizes the primary comparative characteristics of TGS and PTGS. While these pathways share core mechanistic features—including the generation of double-stranded RNA, its subsequent processing into siRNAs by DICER-LIKE enzymes and the assembly of Argonaute-centered ribonucleoprotein complexes—they are functionally and molecularly distinct. These differences manifest in the recruitment of unique protein and enzyme cohorts, the production of specific siRNA size classes (typically 24 nt for TGS and 21–22 nt for PTGS), and the compartmentalization of the silencing machinery. Specifically, the RNA-induced silencing complex is targeted to the cell nucleus for DNA-level repression in TGS, whereas it operates within the cytoplasm to achieve transcript degradation or translational arrest in PTGS. Collectively, these mechanisms constitute a sophisticated plant immune defense system, essentially serving as a “genomic immune system” that maintains genome integrity by preventing the uncontrolled proliferation of transposable elements, viruses, and viroids.

## 5. Leveraging Homology-Dependent Silencing for Targeted Gene Knockdown

The study of mechanisms underlying transcriptional and post-transcriptional silencing of transferred genes and their homologous endogenous plant genes has facilitated the development of methods allowing researchers to knock out the expression of target plant genes, thereby revealing their function in the plant cell and altering cellular metabolism. The following approaches have been successfully used for plant gene knockout.

Brummell et al. proposed incorporating inverted repeats of the 3′ non-coding region of a gene into the genetic construct, where the target gene sequence can be partial. In transgenic tomato plants, it was shown that 91% of primary transformants initiated PTGS of the polygalacturonidase gene; the same strategy with several transcription factor genes was effective in transformed *A. thaliana* plants. The proposed mechanism involves the formation of double-stranded RNA in the 3′ region of the transcribed mRNA, which effectively triggers post-transcriptional silencing of genes homologous to the transgene’s coding region. Since the gene of interest, or only part of it, can be placed into a genetic construct already containing a promoter and the 3′ region in inverted orientation, this method allows for the rapid and efficient analysis of hundreds, or even thousands according to the authors, of plant genes with unknown functions [108].

Introducing heterologous genes into plants within a genetic construct as sense and antisense sequences separated by an intron, which also promotes the transcription of RNA with complementary regions, effectively induces silencing of homologous host genes. Using unique gene sequences allows knocking out the functions of single genes, while using conserved regions enables disrupting the function of multigene families. Using this approach in *A. thaliana*, the function of genes such as *CHS*, *FLC1* (a flowering repressor gene), and *EIN2* (ethylene signaling gene) was disrupted; in cotton plants, genes for Δ12 and Δ9-desaturases were disrupted with a frequency of about 90–100%. The efficiency of the proposed construct was much higher than using sense or antisense constructs with the same genes (0–30%) [109]. Incorporating recombinant genes, including sequences of different genes in sense or antisense orientation, into the genetic construct has been proposed and shown in several studies to enable simultaneous silencing of multiple genes [110,111]. For instance, insertion into tomato plants of a heterologous gene consisting of a 244 bp coding region of the *PG* (polygalacturonidase) gene and a 414 bp region of *PSY* (phytoene synthase, involved in carotenoid biosynthesis) in sense or antisense orientation led to the production of three fruit types: red, yellow, and with spotted coloration. Northern blot analysis showed coordinated suppression of both genes’ expression in yellow fruits [110].

Post-transcriptional silencing of the *FAD2* (Δ9-desaturase) gene in transgenic *A. thaliana* plants, upon introducing genetic constructs promoting the formation of complementary dsRNA, was maintained and inherited over five generations. Thus, the possibility of using this approach for genetic modification of the qualitative composition of fats in seeds was demonstrated [112].

Several studies have documented limitations in the application of siRNA-mediated technologies for host gene knockout. For instance, vectors designed to trigger the production of exogenous siRNAs targeting the promoter regions of seven endogenous rice genes and one reporter transgene successfully induced DNA methylation across all targeted promoters. However, while the transgene was effectively silenced, no transcriptional suppression was observed for the endogenous target genes [113]. Similarly, investigations into the transcriptional silencing of the endogenous GBSSI promoter in potato using inverted repeat (IR) constructs revealed that silencing efficiency varies significantly across different promoter regions. Achieving complete gene repression required the integration of sequences encompassing a full inverted repeat of the promoter into the genetic construct [114].

Despite these challenges, the efficacy of TGS for functional genomics has been demonstrated in maize and wheat. This was achieved by incorporating promoter inverted repeats (pIR) homologous to the anther-specific target genes *Ms45* in maize and *TaMs45* and *TaMs1* in wheat [115,116]. In maize, male-sterile plants were generated with high frequency; their fertility was subsequently restored by expressing the *Ms45* coding region under the control of heterologous promoters [115]. In wheat, the simultaneous silencing of all three homeologs via promoter DNA methylation resulted in male sterility. This approach served as the basis for a dominant male fertility technology for hybrid wheat breeding and seed production, which has been validated under both greenhouse and field conditions [116]. Consequently, TGS represents a robust tool for functional gene analysis in polyploid crops, as a single genetic construct can effectively suppress multiple homeologs across a complex genome through RNA-directed DNA methylation mechanisms.

Silencing of plant gene expression can also be achieved through transient expression of viral vectors in non-transgenic plants carrying a cDNA sequence homologous to plant genes. For this purpose, *Tobacco Mosaic Virus* (TMV) [117], *Potato Virus X* (PVX) [118], *Tomato Golden Mosaic Virus* (TGMV) [119], *Tobacco Rattle Virus* (TRV) [120], and *Tobacco Mosaic Virus Satellite* (STMV) [121] have been proposed. For example, using a two-component system where the STMV serves as a vector for RNA synthesis and TMV as a helper virus effectively induced silencing of 14 different endogenous genes (controlling flower development, leaf development, and biosynthesis pathways of various substances) with a frequency of 60–100% at 10–12 days post-infection [121].

## 6. Ensuring Robust and Stable Transgene Expression: Key Considerations

Transgenic plants are widely used both as genetically modified crops with improved agronomic traits (herbicide resistance, virus resistance, stress tolerance, etc.) [122] and as bioreactors for producing heterologous proteins, including edible vaccines [123], and as model objects for studying plant gene functions and biosynthesis pathways of various enzymes [112]. The use of transgenic plants as commercial crops imposes strict requirements, including the stability of target gene expression. Studying the molecular genetic mechanisms of foreign gene inactivation in various transgenic plant models has identified several factors that increase the likelihood of transgene silencing. This information is crucial for researchers as it allows them to significantly reduce, if not completely eliminate, the risk of losing target gene expression in transformants, subsequent generations, and hybrids from various crosses [124]. Key points are summarized below.

### 6.1. Vector Design Optimization for Gene Silencing Prevention

Factors influencing gene expression levels must be integrated into the initial design phase of plant transformation vectors. To minimize the risk of gene silencing, the inclusion of homologous sequences within the vector should be avoided; specifically, each recombinant gene should ideally be driven by a unique promoter, terminator, and non-coding regulatory regions.

Careful consideration must be given to promoter strength and the anticipated expression level of the target gene, as excessive transcript accumulation can trigger PTGS. This risk is particularly acute when the genome contains an endogenous gene with high homology to the transgene. Achieving an optimal expression balance remains a challenge that often requires empirical validation. If gene repression occurs, one potential strategy is the use of a weaker promoter to maintain transgene expression below the silencing threshold. This correlation was first established in early PTGS studies: the use of a double 35S promoter led to *CHS* gene co-suppression in the majority of transgenic plants (66–81%), whereas constructs utilizing less active promoters (e.g., minimal 35S or the petunia *ChsA* promoter) induced silencing significantly less frequently, in only 2–11% of cases. Consequently, the use of regulatory elements to boost expression requires rigorous preliminary testing to avoid unintended repression [125]. High levels of transgene expression can result in mRNA processing errors and the accumulation of aberrant transcripts, which are subsequently recognized and targeted by the RNA interference machinery [126].

The efficiency of transcription termination is a critical factor in preventing the formation of aberrant mRNAs and the subsequent induction of PTGS. A comparative analysis of the tHSP and T35S terminators revealed that even highly efficient terminators cannot always fully preclude promoter methylation. However, the use of tHSP has been shown to yield higher expression levels and a reduced degree of DNA methylation within the reporter gene promoter [127]. Recent investigations into the contribution of various regulatory elements to transgene stability reinforce the central role of terminators. The tHSP and tNOS terminators have been identified as the most effective, enhancing mRNA stability, *gfp* expression levels, and splicing efficiency [128]. Consequently, genetic constructs should incorporate robust terminators to prevent the formation of unpolyadenylated transcripts, which serve as substrates for siRNA synthesis. Furthermore, the inclusion of Matrix Attachment Regions (MARs) flanking the vector can mitigate “read-through” by RNA polymerase beyond termination sites. This prevents the synthesis of aberrant antisense RNAs originating either from the transgene itself or from adjacent endogenous plant promoters [124,129].

Typically, recombinant genes consist solely of a coding sequence (cDNA) lacking introns and are driven by viral or bacterial promoters. The absence of characteristic plant regulatory elements often renders such constructs “foreign” to the host genome, potentially triggering host defense mechanisms. Research indicates that transgenes structurally analogous to endogenous plant genes—incorporating native promoters, 5′-UTRs, introns, 3′-UTRs, and terminators—exhibit significantly higher resistance to both transcriptional and post-transcriptional gene silencing [130,131].

The inclusion of inverted repeats or truncated gene copies within the genetic construct is highly detrimental. These structures can lead to the synthesis of aberrant RNAs, which serve as substrates for siRNA biogenesis and activate the RNA interference machinery. Furthermore, the presence of extensive vector backbone sequences should be minimized, as they are known to frequently provoke de novo DNA methylation. In the presence of secondary siRNAs, this methylation can spread to adjacent regions, including the target transgene.

The methodology of synthetic biology, focused on optimizing the design of genetic elements to ensure stable transgene expression, is discussed in detail in recent comprehensive reviews [132,133].

### 6.2. Insulating Transgenes from Position Effects and Genomic Context

Integration of genetic constructs into the plant genome using traditional transformation methods typically occurs randomly, contributing to the wide variability of expression among independently obtained primary transformants [22]. To reduce the influence of the surrounding genomic context on the stability of heterologous gene expression, homologous and site-specific integration into a defined chromosomal region is used. Methods for introducing heterologous genes using site-specific recombination involve systems like the *Cre*-lox system from bacteriophage *P1* and the *FLP*/*FRT* system from yeast, which are active in plant cells. This method allows not only for the introduction of mutations into plant genes but also for placing the transgene in a specific genomic location, enabling control over target gene expression [14,134]. The CRISPR/Cas9 system is now most widely used for site-specific integration, significantly expanding researchers’ capabilities by allowing gene insertion into virtually any region of the plant genome. Approaches are being developed to find the most favorable genomic regions (“safe harbors”) that would ensure high levels of transferred gene expression without affecting the plant’s own genes [135].

Another strategy to ensure stable transgene expression independent of the integration site is the inclusion of additional fragments—MAR elements—flanking the target genes, which are thought to promote the formation of an active chromosomal domain [136]. However, data on their effect on the stability of foreign gene expression are conflicting. Introducing MAR elements from the tobacco *Rb7* gene into the genetic construct increased the expression level of the *uidA* gene in transformants with a low copy number of foreign insertions but had no effect in the presence of multiple tandem insertions [137]. An experiment conducted in Vaucheret’s lab involved crossing tobacco plants with a 35S-*uidA* transgene containing various types of MAR elements (from chicken, yeast, tobacco, and bean genes) with tester lines, causing trans-inactivation (locus 271) or post-transcriptional silencing (locus 6b8) of other transgenes. Analysis of *uidA* gene expression in hybrids showed that the presence of MAR elements did not prevent reporter gene inactivation [138]. Another research group, similarly to the previous study, showed that the presence of the MAR element from the tobacco *Rb7* gene did not prevent trans-inactivation-type interactions in hybrids when crossed with a line carrying the silencer gene 271. However, hybridization with lines C40 and C190, which also exhibit silencing properties for sequences under the control of the 35S promoter but to a lesser extent, revealed a protective role of the MAR element in some transformants [139]. Mlynarova et al. showed that in transgenic tobacco plants obtained by site-specific recombination, the presence of a MAR element from the chicken lysozyme A gene promoted a high level of β-glucuronidase expression. Removal of the MAR element from the 5′ region of the *uidA* gene led to post-transcriptional silencing of transgene expression in homozygous progeny for two transformants [129,140].

Transgenic tobacco plants harboring constructs flanked by tobacco-derived TM2 MARs and the M4 insulator exhibited significantly enhanced expression levels of the PHB-01 antibody compared to those transformed with a conventional vector [141]. These findings underscore that incorporating MAR sequences and insulators can effectively bolster both the magnitude and stability of transgene expression by mitigating repressive position effects. However, further systematic investigations are required to elucidate the long-term epigenetic stability conferred by these elements across diverse plant taxa and integration sites.

### 6.3. Control of Transgene Copy Number and Integration Patterns

Extensive experimental evidence indicates that multiple insertions and tandem repeats within the integration site often trigger transgene silencing mechanisms. Therefore, the selection of single-copy integration events is highly preferred during the screening of primary transformants. Molecular validation using Southern blot hybridization or quantitative PCR is essential to confirm T-DNA integrity and ensure the absence of rearranged fragments or vector backbone sequences. Modern approaches, such as digital PCR (dPCR) and Oxford Nanopore sequencing, provide enhanced precision in resolving complex integration landscapes [133,142].

The frequency of foreign DNA integration—whether at single or multiple loci—is significantly influenced by the transformation method. *Agrobacterium*-mediated transformation remains the gold standard for achieving stable expression, as it yields single-copy insertions in approximately 50% of cases. In contrast, biolistic transformation (particle bombardment) frequently results in high copy numbers and complex genomic rearrangements. While the choice of transformation system is often constrained by the recalcitrance of specific plant species to *Agrobacterium* or the availability of in vitro regeneration protocols, the potential for complex integration must be carefully managed.

Furthermore, transitioning transgenes to a homozygous state can occasionally lead to loss of expression via PTGS, even in lines where the hemizygous parent showed stable activity. Consequently, rigorous monitoring of expression stability is mandatory across both hemizygous and homozygous progeny to ensure the long-term performance of the transgene [124].

### 6.4. Optimization of Cultivation Conditions for Transgene Expression Stability

The stability of transgene expression is significantly influenced by environmental factors and cultivation conditions, affecting both cell suspension cultures and intact transgenic plants. For instance, thermal stress has been shown to drastically increase silencing frequency: in alfalfa cell cultures, a 10-day treatment at 37 °C resulted in a 95% loss of herbicide resistance gene expression, compared to only 12% after 150 days of cultivation at the standard 25 °C [143].

The transition from the sterile, constant conditions of in vitro systems to the fluctuating environment of the field can trigger substantial changes in recombinant gene activity. In a large-scale field trial involving 38 homozygous transgenic tobacco lines, the frequency of nitrate reductase transgene inactivation varied significantly between independent events, reaching up to 57% [144]. Similarly, in transgenic petunia, the frequency of the “white/mosaic flower” phenotype caused by *A1* transgene silencing rose from approximately 5% in greenhouse settings (*n* = 2110) to 60% under field conditions (*n* = 30,000) [145]. Further studies, utilizing reporter genes such as *gfp* in transgenic rapeseed, confirm that expression variability is highly dependent on the cultivation environment, showing marked differences between controlled growth conditions, greenhouses, and field plots [25]. Consequently, the development of GM crops requires rigorous screening and multi-location field testing. This process is essential to select resilient lines where transgene expression remains robust and independent of external environmental fluctuations.

### 6.5. Exploiting Silencing-Deficient Mutants for Enhanced and Stable Heterologous Expression

An alternative strategy to ensure robust transgene performance involves utilizing host plant mutations that suppress endogenous gene silencing pathways [124,146]. For instance, stable and high-level expression of the *uidA* reporter gene was achieved in *A. thaliana* mutants deficient in silencing components, such as *sgs1* or *sgs3*. Notably, the integration of a chicken lysozyme MAR into the construct synergistically enhanced expression: *uidA* activity increased 5-fold in the *sgs1* background and 12-fold in the *sgs3* background compared to transformants without mutations [147].

Furthermore, evidence suggests that directed selection can be employed to stabilize specific expression phenotypes in subsequent generations, allowing for the fixation of either high or low expression levels [4,124].

In the context of modern plant biotechnology, these findings support a multifaceted approach to transgene management. By combining optimized vector design, the use of specialized host genetic backgrounds, and rigorous environmental screening, researchers can overcome the inherent challenges of epigenetic instability to develop high-performance transgenic crops.

## 7. Conclusions

The breakthroughs in understanding the molecular and genetic mechanisms of transgene silencing, hailed as revolutionary over two decades ago [148], continue to drive intensive research into the complexities of epigenetic modifications [57,149]. Modern studies are revealing novel roles for long-established enzymes and proteins. For instance, in pollen sporophytic tissues, RNA Pol IV has been shown to primarily mediate post-transcriptional gene regulation through the biogenesis of 21–22 nt siRNAs, a process potentially linked to the establishment of hybridization barriers [150]. Today, it is unequivocally recognized that post-transcriptional gene silencing serves as a robust immune defense against viruses and viroids, while transcriptional gene silencing acts as a critical safeguard against the uncontrolled proliferation of transposable elements. The RNA-directed DNA methylation pathway provides plants with the plasticity required to modulate endogenous gene expression during development [151] and in response to fluctuating environmental conditions and biotic stressors [152]. Furthermore, RdDM is vital for proper endosperm development, balancing maternal and paternal genomic contributions [153]. Current debates also highlight the pivotal role of RdDM in paramutation, genomic imprinting, and intercellular signaling [154]. Finally, the continuous refinement of RNAi technologies offers innovative strategies for enhancing disease resistance and ensuring sustainable agricultural yields in an era of global climate change [155].

## 8. Applications and Future Perspectives

In the current agricultural landscape, plant breeders face a complex new challenge: developing high-yielding cultivars that are not only adapted to specific agroecological conditions but also possess the plasticity to respond rapidly to emerging biotic and abiotic stresses. Understanding and harnessing natural epigenetic regulatory mechanisms is becoming pivotal to achieving this goal. For instance, in the Solanaceae family, particularly in tomato (*Solanum lycopersicum*) and potato (*Solanum tuberosum*), critical developmental processes such as floral transition, fruit ripening, and tuberization are governed by sophisticated epigenetic landscapes. Furthermore, numerous agronomically significant epialleles have been identified across diverse crops, including the dwarf phenotype in rice, enhanced anthocyanin production in apple, modified oil content in oilseed rape, high heterosis in pigeon pea, and sex determination in melon [156,157]. Modern researchers can now generate novel epigenetic variants by modulating the activity of key epigenetic enzymes or by employing precision epigenome editing tools. These approaches allow for the targeted delivery of regulatory factors to specific genomic loci, facilitating the creation of “designer” crops with enhanced environmental resilience and optimized yield traits.

### 8.1. Practical Applications of Epigenetic Modulation

For instance, RNAi-mediated repression of the *DDM1* gene in poplar resulted in increased resistance to drought-induced cavitation [158]. Similarly, VIGS-induced silencing of the *MET1-like1* gene in pepper (*Capsicum annuum*) led to DNA hypomethylation and accelerated fruit ripening. This phenotypic shift was characterized by increased soluble solids content, carotenoid accumulation, cell wall degradation, elevated abscisic acid (ABA) levels, and a concomitant reduction in indole-acetic acid (IAA) [159]. Furthermore, in rice (*O. sativa*), susceptibility to *Rice Black-Streaked Dwarf Virus* (RBSDV) was found to be enhanced by the overexpression of *OsAGO2*, a key component of de novo methylation. Conversely, *ago2* mutant lines exhibited robust viral resistance by triggering early defense responses, including the upregulation of defense-related genes and the production of reactive oxygen species (ROS) [160].

### 8.2. Large-Scale Screening of Epigenetic Effectors for Targeted Silencing

Components of the RdDM pathway are increasingly utilized to activate or inactivate endogenous genes through site-specific DNA methylation and histone modifications, which can be stably inherited across multiple generations [161,162]. For precision epigenome editing, technologies such as Zinc-finger DNA-binding domains and CRISPR/Cas9 systems utilizing catalytically inactive “dead” Cas9 (dCas9) have been developed. When fused with effector proteins—such as DNA methyltransferases or demethylases—these tools enable precise, programmable epigenetic modifications at specific genomic loci, facilitating the creation of stable, non-transgenic phenotypic variations.

Leveraging synthetic biology approaches—specifically the fusion of ZF proteins with individual RdDM components in *Arabidopsis* silencing mutants—researchers have identified the most potent factors for inducing ectopic DNA methylation and target gene silencing. Among nine evaluated factors, the DMS3 component (part of the DDR complex) proved most effective. DMS3 actively recruits Pol V, AGO4, AGO6, and AGO9, successfully inducing *FWA* gene methylation and silencing even in the *nrpd1* mutant background, where Pol IV deficiency typically abolishes siRNA biogenesis. Additionally, the MORC6-ZF fusion was identified as a highly efficient tool for inducing silencing. However, genome-wide analysis revealed a significant constraint: while ZF-mediated recruitment of RdDM components occurred at thousands of non-target sites, only a small fraction produced siRNAs and underwent directed DNA methylation. This suggests that the mere recruitment of Pol V and AGO is insufficient to activate the full RdDM pathway at most genomic loci. Notably, the simultaneous targeting of Pol IV and Pol V activities—achieved through a combination of NRPD1-ZF and DMS3-ZF fusions—synergistically enhanced efficiency, leading to the methylation of thousands of loci. These findings underscore that future strategies for effective epigenome modulation must integrate factors involved in both siRNA biogenesis and Pol V activity. Furthermore, these principles established with ZF platforms are directly applicable to other DNA-binding systems, such as CRISPR/dCas9 [163].

In a comprehensive screening of 270 different proteins involved in epigenetic signaling and chromatin remodeling in *Arabidopsis*, researchers utilized the *fwa* epiallele as an endogenous reporter system. In its unmethylated state, *fwa* results in a characteristic late-flowering phenotype, providing a clear visual marker for silencing efficiency. This study identified 14 potent effectors capable of repressing *fwa* expression through diverse molecular pathways, including the establishment of de novo DNA methylation, induction of H3K27me3 marks, H3K4me3 demethylation, and the deacetylation of H3K9, H3K14, H3K27, and H4K16. Furthermore, several identified proteins acted by inhibiting Pol II transcriptional elongation or through Pol II dephosphorylation. These effectors successfully restored the early-flowering phenotype of the *fwa* epiallele to wild-type levels. Notably, key components of the RdDM pathway—including DMS3, SUVH2, SUVH9, and MORC1—were among the most effective silencing factors [164]. These findings significantly expand the molecular toolkit available for precision epigenome engineering, demonstrating that effective gene repression can be achieved through multiple, non-redundant epigenetic modifications beyond simple DNA methylation.

Recent studies have demonstrated the potential of using the catalytic domain of the histone methyltransferase SDG2 (SDG2cd) fused with ZF domains to direct the tri-methylation of histone H3 lysine 4 (H3K4me3). As a highly conserved chromatin mark associated with transcriptionally active regions, H3K4me3 acts as a potent anti-DNA methylation signal. The SDG2cd–ZF complex has been shown to effectively abolish DNA methylation at the *FWA* locus and eliminate CG methylation across numerous other genomic regions in *Arabidopsis* by recruiting endogenous DNA demethylases [165]. Furthermore, the CRISPR-based SunTag system has been successfully employed to recruit H3K4me3 methyltransferases for precise gene activation. Beyond the induction of *FWA* expression, the SunTag-SDG2 platform was utilized to enhance pathogen resistance by targeting the H3K4me3-dependent disease resistance gene *SNC1*. This gene, which encodes a nucleotide-binding leucine-rich repeat (NLR) protein, is located within the partially silenced *RPP5* gene cluster. Epigenetically edited *Arabidopsis* plants exhibited significantly increased resistance to the hemibiotrophic pathogen *Pseudomonas syringae* pv. tomato DC3000 [166]. These results highlight the efficacy of targeted histone modifications in reprogramming plant immunity and overcoming epigenetic repression of defense-related loci.

The aforementioned studies demonstrate the promising potential of epigenome editing for the precise activation or repression of endogenous gene expression. However, further research is required to refine DNA-binding specificity and minimize off-target effects across the genome. A significant challenge remains in ensuring the long-term, transgenerational stability of induced epigenetic marks, as some modifications currently exhibit reversibility over successive generations.

### 8.3. Future Perspectives: From Proof-of-Concept to Crop Improvement

Despite these hurdles, modifying the epigenetic landscape offers vast potential for crop improvement. To date, practical applications in major crops are emerging; for instance, researchers have developed edited cassava (*Manihot esculenta*) lines resistant to *Cassava Bacterial Blight* (CBB). Pathogens often exploit the expression of host susceptibility (*S*) genes to facilitate infection. By blocking access to these *S* genes through targeted promoter methylation—utilizing a synthetic ZF domain fused to the RdDM component DMS3—scientists produced plants with significantly attenuated disease symptoms while maintaining normal growth and development [167].

Furthermore, several candidate genes have been proposed for enhancing key agronomic traits. For example, the epigenetic downregulation of *GW2*, *GW5*, or *TGW*6 in rice is predicted to increase grain size. Conversely, nutritional fortification could be achieved through the epigenetic activation of the nicotianamine synthase (*NAS*) gene family or the carotenoid biosynthetic pathway in the endosperm [168].

### 8.4. Exploiting Transposon-Derived Anti-Silencing Factors

Recent studies have characterized a novel class of anti-silencing proteins, designated VANC, which are encoded by the VANDAL family of transposable elements. These proteins specifically target and remove DNA methylation at their cognate loci to maintain their own transcriptional activity [169]. The discovery of VANC-mediated demethylation mechanisms opens a compelling frontier for plant biotechnology. In the future, these proteins could be engineered as molecular tools to counteract transcriptional gene silencing or to reactivate silenced transgenes in previously methylated lines. By incorporating VANC-like factors into vector designs, researchers may develop self-sustaining systems that actively prevent the accumulation of repressive epigenetic marks, thereby ensuring the long-term stability and robust expression of recombinant genes.

### 8.5. Regulatory Landscapes and Future Perspectives for Epigenome-Edited Crops

In the current global context of 2025–2026, the legal status of plants derived through epigenome editing remains a subject of intense scientific and juridical debate. While the EU GMO legislation dates back to 2001, recent technological advancements have prompted a re-evaluation of these frameworks, as current rules often apply GMO-level restrictions to all edited plants [170]. The core of the debate is whether targeted epigenetic modifications—which introduce neither foreign DNA nor mutations into the primary sequence—should be classified as “genetic modifications.”

A landmark shift occurred on December 4, 2025, when the European Union reached a provisional agreement on a new regulatory framework for plants derived from New Genomic Techniques (NGTs). Under this framework, plants with modifications that could occur naturally or through conventional breeding (classified as NGT1) will no longer be subject to the full GMO authorization process. In contrast, more complex edited plants fall into the NGT2 category and remain under existing GMO regulations. This agreement awaits formal approval by the European Parliament and the Council in early 2026.

Globally, regulatory approaches are diversifying. Since 2022, China has implemented streamlined rules, reducing approval timelines for new breeding techniques (NGTs) to 1–2 years, though pre-market assessments remain similar to GMO protocols. India has exempted products developed via SDN1 and SDN2 protocols from GMO classification, provided they are free of foreign DNA. In Africa, nations including Burkina Faso, Ethiopia, Kenya, Nigeria, and Malawi are adopting adaptive, case-by-case regulatory frameworks based on risk proportionality [170]. Detailed analyses of these shifting paradigms are available in recent comprehensive reviews [170,171]. Ultimately, epigenome editing emerges as a transformative tool for “New Breeding Techniques,” potentially offering a pathway to circumvent the stringent regulatory barriers associated with the “GMO” designation while delivering next-generation crop solutions.

## Figures and Tables

**Table 1:** Structure and location of T-DNA insertions in the tobacco plant genome.

Line	Copy Number	Vector DNA	Chromosomal Location	Expression
H9	1	-	Telomeric	Stable, silencing-resistant [47]
Kα	1	-	n.d	Stable, silencing-resistant [48]
H83	2 (one of which has rearrangements)	+	Telomeric	Stable, silencing-resistant [47]
K81	1	+	Telomeric	Stable, silencing-susceptible [48]
H59	1	+	Intercalary	Unstable [47]
H9np	2 (inverted duplication)	n.d	n.d	Unstable, silencer loci [49]
H11	2–3 (rearrangement)	+	Pericentromeric	Unstable, silencer loci [47]
H2	4 (closely linked copies with an inverted duplication)	+	Close to intercalary heterochromatin	Unstable, silencer loci [48]
271	6–7	n.d	Telomeric	Unstable, silencer loci [35]

n.d—not determined.

**Table 2:** Comparative analysis of TGS and PTGS gene silencing mechanisms in plants.

Main Characteristics	TGS	PTGS
Main enzymes required for siRNA formation	PolIV	PolII
	RDR2/6	RDR6
	DCL3	SGS3
	HEN1	DCL2/4
	AGO4	HEN1
		AGO1
siRNA size	24–26 nt	21–22 nt
Location of the AGO-siRNA ribonucleoprotein complex	Nucleus	Cytoplasm
siRNA target	Gene promoter region	mRNA
Consequences	Inactivation of gene/transgene expression at the transcriptional level. Methylation of target DNA in the promoter region, histone modifications.	Inactivation of gene/transgene expression at the posttranscriptional level. Translation arrest or mRNA degradation. Methylation of the coding region of the target gene in case of secondary siRNA formation.
Inheritability	Inherited across generations.	Not heritable.
Biological role	Suppresses the activity of repetitive DNA elements and transposable elements. Defense mechanism against DNA virus infection.	Plant viral defense.

## Data Availability

No new data were created or analyzed in this study. Data sharing is not applicable to this article.
